# An Evaluation of the Treatment of Full-Thickness Wounds Using Adipose Micro-Fragments within a Liquid Dermal Scaffold

**DOI:** 10.3390/ebj3030040

**Published:** 2022-09-17

**Authors:** Sara Sheikh-Oleslami, Ida Hassanpour, Nafise Amiri, Reza Jalili, Ruhangiz Taghi Kilani, Aziz Ghahary

**Affiliations:** 1 Department of Medicine, University of British Columbia, Vancouver, BC V6T 1Z4, Canada; 2 International Collaboration on Repair Discoveries (ICORD), Vancouver, BC V5Z 1M9, Canada; 3 Division of Plastic Surgery, Department of Surgery, University of British Columbia, Vancouver, BC V6T 1Z4, Canada

**Keywords:** extracellular matrix, collagen, scaffold, wound healing, adipose

## Abstract

In full-thickness wounds, inflammation, lack of matrix deposition, and paucity of progenitor cells delay healing. As commercially available solid (sheet) scaffolds are unable to conform to wounds of varying shapes and sizes, we previously generated a nutritious, injectable, liquid skin substitute that can conform to wound topography. In combination with adipose micro-fragments as a viable source of progenitor cells, a composite, in situ forming skin substitute was tested for the treatment of silicon ring splinted full-thickness wounds in rats. The in vitro survivability and migratory capacity of adipocytes derived from rat micro-fragmented fat cultured in our scaffold was examined with a Live/Dead assay, showing viability and migration after 7 and 14 days. In vivo, the efficacy of our scaffold alone (LDS) or with adipose micro-fragments (LDS+A) was compared to a standard dressing protocol (NT). LDS and LDS+A showed ameliorated wound healing, including complete epithelialization and less immune cell infiltration, compared to the NT control. Our findings demonstrate that a 3D liquid skin scaffold is a rich environment for adipocyte viability and migration, and that the addition of adipose micro-fragments to this scaffold can be used as a rich source of cells for treating full-thickness wounds.

## 1. Introduction

While wound healing is essential for tissue homeostasis, it is a complex and dynamic process involving precise and overlapping exudative, inflammatory, proliferative, and extracellular remodeling phases, all of which involve cell–cell interactions as well as cell–matrix signaling [1,2,3,4]. This intricate physiological process involves a multitude of cell strains and their products [1,2]. Chronic wounds fail to follow this sequential series of events in order to restore tissue homeostasis [3], often delayed in the proliferative stage due to high levels of inflammation, delayed matrix deposition, and colonization or delayed clearance of bacteria [4]. The longer a wound remains open, the greater the risk of infection which can degrade the deposited matrix within the wound [4,5]. These infections often recur, lasting many years, some of which possibly gain antibiotic resistance [5].

These chronic, non-healing wounds, such as burns, lacerations, ulcers (diabetic, arterial, pressure), and infectious or surgical wounds pose a serious threat to healthcare systems globally [6]. It is estimated that 1–2% of the general population in developed countries will experience a chronic wound in their lifetime, often compounded by other comorbidities such as diabetes or obesity [7]. This comes at a great financial burden to the health care system, with an estimated $28.1–96.8 billion USD spent annually on treatment in the United States alone [8,9]. Moreover, the quality of life in patients with chronic wounds is greatly decreased [7], and therefore, it is crucial to find an effective strategy to induce rapid closure of the wound, effectively reducing healthcare costs and improving patient outcomes, including reducing the risk of progression to a chronic wound, scar formation, or infection [10].

One strategy for tissue repair and regeneration is the use of biomimetic scaffolds that foster the growth and development of tissue towards restoring normal architecture. One major problem with commercially available solid (sheet) scaffolds of uniform thickness is their inability to conform to wounds of varying shapes and sizes, which is particularly challenging when dealing with deep ulcers [10]. In fact, the presence of such cavities and void spaces within deep ulcers makes these wounds very difficult to heal with conventional methods.

To overcome this problem, a liquid and flowable skin substitute is required which can fill the irregular cavities and tunnels commonly observed in deep ulcer wounds. To address this, our lab has generated a prototype of an injectable skin substitute which is a cross-linked, collagen-glycosaminoglycan-based scaffold with polyvinyl alcohol (PVA)-borate. It is a powdered, shelf-ready product that can be reconstituted, and is able to rapidly solidify at 35–37 °C [11,12]. It is also more resistant to specific degradation by collagenases compared to standard collagen scaffolds due to the entanglement of PVA-borate networks along collagen fibrils, as well as the crosslinking of the collagen itself [11]. Compared to other commercially available products, this liquid dermal scaffold (LDS) can fill and conform to wounds of any shape and depth from the bottom-up. It also has all the necessary ingredients for skin cells to be nourished, proliferate, and migrate in.

The use of biomaterials, either natural or synthetic, in the reparation, replacement, or enhancement of tissues or organs is gaining traction in the medical field due to the myriad applications they have, whether it be orthopedic implants, cardiac stents, urinary catheters, or for wound healing [13,14,15].

The initial introduction of such materials demonstrated many issues, including tissue reactivity and biocompatibility [16]. With advancements in the field, the use of such materials in medical practice has become more commonplace. Specifically, collagen-based biomaterials, which first arose in the 1900s, have now changed how soft-tissue wounds are managed [17,18]. This is largely due to the functions of collagen within the extracellular matrix (ECM), providing organization, stability, and tensile strength to healing tissues [18,19]. Moreover, collagen is non-toxic and biodegradable, and thus less likely to cause tissue reactivity when applied [18,19]. A broad review of the existing literature, however, has yielded no reports of a liquid collagen-based scaffold for the treatment of full-thickness wounds.

Another factor hindering the healing of full-thickness, chronic wounds is the paucity of progenitor cells [4]. As such, the addition of a viable source of progenitor or stem cells to the wound bed is considered a plausible strategy to improve the healing process [20,21]. Adipose tissue is a rich source of mesenchymal stem cells which has recently been widely used for regenerative or cosmetic purposes [21,22,23]. However, it has been well documented that mesenchymal cells, including adipose-derived cells, need an extracellular matrix to survive and function normally [24].

Here, we used a murine model of excisional wound healing [25] to demonstrate that combining adipose tissue micro-fragments into the LDS to develop a composite, in situ forming skin substitute will ameliorate the healing of full-thickness wounds.

## 2. Materials and Methods

### 2.1. Liquid Dermal Scaffold

A crosslinked collagen–glycosaminoglycan (GAG)-based liquid dermal scaffold containing polyvinyl alcohol (PVA) hydrogel was used as the LDS. The contents, preparation, mechanical properties, and functionality were previously described in detail [11,12]. Briefly, type 1 bovine collagen (Advanced Biomatrix, Carlsbad, CA, USA) and chondroitin-6-sulfate (Sigma Aldrich, St. Louis, MO, USA) were combined in a 1:6 *w*/*w* ratio to a concentration of 3 mg/mL collagen and neutralized with DMEM and 1N NaOH [12]. Glutaraldehyde (0.02% *v/v*; Sigma Aldrich) was used to crosslink the collagen, then glycine was used to deactivate residual aldehydes [12]. After washing, hydrogels comprising PVA (50:50/208,000 and 146,000 MW, 0.2% *w/v*; Sigma Aldrich), sodium borate decahydrate (0.05% *w/v*; Sigma Aldrich), and ascorbate (pH 7, 100 mM; Sigma Aldrich) were added to the crosslinked collagen [12]. The scaffold was maintained at 4 °C in liquid form until use [12].

### 2.2. Combining Adipose Micro-Fragments into the LDS

All protocols for this experiment were conducted in accordance with protocols approved by the University of British Columbia (B20-0022) and the Canadian Guidelines on Animal Care. All procedures followed the aseptic surgical technique and all animals received humane care throughout the course of the experiment. All rats were anesthetized with isoflurane and euthanized by CO_2_ asphyxia [10].

Subcutaneous inguinal adipose tissue was excised from one rat to obtain adipose micro-fragments with a modified protocol of what was previously described [10,26] and stored at 4 °C. The adipose tissue was washed with phosphate buffer saline (PBS pH 7.4; Sigma Chemical Co, St. Louis, MO, USA) with 3× (300 µg/mL) streptomycin and 3× (300 µg/mL) penicillin at room temperature (25 °C) for a total of 15 min, with the adipose tissue being placed in a new PBS wash every five minutes. The tissues were minced to obtain fragments of approximately 1 mm in length and width. The LDS and adipose micro-fragments were then combined in a ratio of 9:1 into a microtube and mixed thoroughly. Then 900 µL LDS and 100 µL adipose tissue fragments were added into a microtube and mixed thoroughly. These combinations were then added into wells of a 12-well plate which was subsequently placed in an incubator at 37 °C and 5% CO_2_ for 30 min to allow for the gel combinations to settle. Next, 2 mL Dulbecco’s modified eagle’s medium (Hyclone DMEM/High Glucose; GE Healthcare, Logan, UT, USA) supplemented with fetal bovine serum (FBS 10% *v/v*; Gibco, Grand Island, NY, USA) and antibiotic/antimycotic (1% *v/v* Anti-Anti 100×, Gibco) was added onto each gel combination and incubated again for 7 or 14 days. Throughout this time, the medium was changed every three days.

### 2.3. Cell Viability and Migration Assay

Cell viability and migration were assessed on days 7 and 14 with a Live/Dead toxicity assay using the LDS and adipose tissue gel combinations made prior. The DMEM medium was first removed from the wells, and then each well was washed three times with 1× PBS (pH 7.4) supplemented with antibiotic/antimycotic (1% *v/v* Anti-Anti 100x, Gibco). Half of the wells were treated with 1.5 mL 70% ethanol and incubated for 30 min to serve as a negative control. The Live/Dead assay was conducted in accordance with the instructions detailed by the manufacturer (Live/Dead Double Staining kit, Calbiochem, Gibbsstown, NJ, USA). The 12-well plate was then incubated for 45 min. Following incubation, the dye was removed and 1 mL 1× PBS was added to each well and visualized using a Zeiss Axiovert microscope and Axiovision 4.8 software to capture images set to detect FITC and Texas Red.

Assessment of cell viability was done by comparing treatments with Calcein-AM, EthD1, and a Calcein-AM-EthD1 overlay. The treatment wells were stained with Calcein-AM and a Calcein-AM-EthD1 overlay to see the number of live cells in the system, as well as the amount of live and dead cells in the system, respectively. The negative control was stained with EthD1 only to determine whether dead cells were present in the system following a 70% ethanol wash.

Assessment of cell migration was qualitative, comparing images on days 7 and 14 to see if the images remained static or whether there was the migration of cells outwards from the adipose micro-fragments.

### 2.4. Animal Model and Wound Generation

Using a previously described model of excisional wound healing with some variations [25,27], 7 Crl:CD(SD), rats were purchased from Charles River Laboratories (Wilmington, MA, USA). Two rats were housed per cage prior to wounding and fed an unrestricted amount of rodent chow and water. All cages were kept in standardized conditions at an institutional animal facility with a 12 h light–dark cycle. The rats were housed in these conditions during both the acclimation and experimental periods (1 and 2 weeks, respectively). After wounding, all rats were weighed, and two rats were housed per cage.

All wounding and post-operative care were performed by one surgeon. The rats were weighed prior to the procedure and then anesthetized with 2% isoflurane in 100% oxygen at a flow rate of 1.0 L/min for the entirety of the surgical procedure. Two sterile silicone splints (Grace Bio-Labs, Bend, OR, USA) with an inner and outer diameter of 8 and 12 mm, respectively, were placed around the pre-designated parathoracic wound area and secured using six outer single interrupted sutures (4-0 Prolene Nylon Suture, Ethicon LLC, Cornelia, GA, USA).

Two full-thickness, 8 mm wounds, one on either side of the midline were created on the shaved dorsal hemithorax of each rat using an 8 mm biopsy punch tool (Acuderm, Fr. Lauderdale, FL, USA). The surgical procedure detailed has previously been described with slight adaptations [10,12,25,28]. A semi-occlusive dressing (Tegaderm, 3M, St. Paul, MN, USA) was subsequently applied to the wound. Following the procedure, two rats were housed per cage, placed on a warm water circulation veterinary blanket, and observed until full recovery from the anesthesia.

### 2.5. Experimental Design

**Figure 1:**
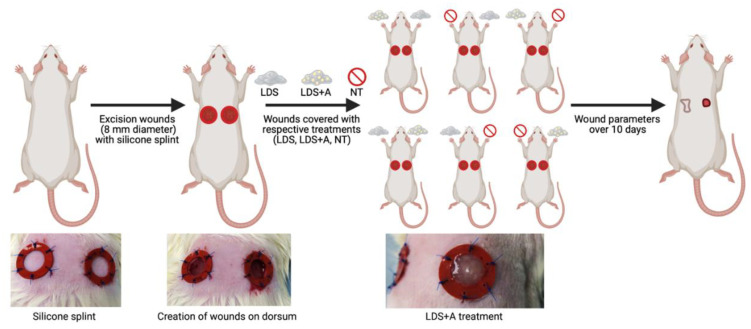
Graphical representation of experimental design. Created with Biorender.com (accessed on 5 August 2022).

Upon wounding, six rats (12 wounds total) were randomly assigned one of three treatment conditions which were defined as follows: Group A: liquid dermal scaffold + adipose micro-fragment combination (LDS+A) vs. no treatment (NT); Group B: liquid dermal scaffold only (LDS) vs. no treatment (NT); Group C: liquid dermal scaffold + adipose micro-fragment combination (LDS+A) vs. liquid dermal scaffold only (LDS) (Figure 1). One rat was used as a fat donor throughout the experiment.

### 2.6. Treatment and Postsurgical Evaluation

Adipose tissue was extracted from the inguinal region of the donor rat and kept at 4 °C [10,26]. Following the protocol described above, for the LDS treatment, thoroughly mixed 9:1 combinations of LDS and adipose micro-fragments were made. Then 250 µL of this combination was applied to the wound bed in an even distribution. The LDS-only group received 250 µL LDS without any adipose micro-fragments. The scaffolds were maintained in liquid form on ice prior to wound generation, then removed and left to sit at 25 °C for five minutes prior to administration. All wounds were covered by semi-occlusive dressing (Tegaderm, 3M, St. Paul, MN, USA). A layer of sterile gauze was then placed to absorb extra pressure followed by a secondary layer of dressing (3M Coban) over the Tegaderm dressing to prevent splint removal by the rats and interference with the wound healing process. The NT group received dressing only. Treatments were monitored every three days throughout the experiment. The general health condition and weight of the rats were monitored daily according to protocols. The rats were anesthetized to assess the wound beds on days 0, 3, and 6 during bandage replacement for splint stability, inflammation, and signs of infection. The wound bed was also imaged during this time using a digital camera. All rats were euthanized on day 10. Using a 12-mm circular biopsy punch, all wound sites were then harvested for tissue analysis.

### 2.7. Planimetry Analysis of Wound Closure Rates

Photographs were analyzed to determine the percentage of wound closure using image analysis software (ImageJ, National Institutes of Health, Bethesda, MD, USA). All image analyses were performed by two blinded reviewers. The wound margins were traced and the percent wound closure area at each time point was calculated in pixels using the following equation: % Wound closure = 100% − (open wound area/original wound area) × 100% [10]. From this, the rate of wound closure was derived. The wound sizes were normalized as the splints used had a constant area.

### 2.8. Histological Analysis

Wound sites were harvested and the tissue samples were fixed in 10% formalin for 24 h and embedded in paraffin blocks as previously described [29]. Then 5 µm tissue sections were stained with Hematoxylin and Eosin and Masson’s Trichrome (MT) staining using standard protocol [29]. Slides were studied and imaged using a Nikon Eclipse 80i microscope (Nikon Corporation, Tokyo, Japan). The sections were analyzed using Image J software (National Institute of Health, Bethesda, MD, USA).

For each section, a randomized area (magnifications 2× and 10×) was photographed and assessed for other parameters using the digital analysis software including epidermal thickness as well as collagen deposition and organization. All assessments were conducted blindly by two reviewers.

**Epidermal Thickness:** Using ImageJ, the epidermal thickness (ET) of the histology sections was calculated as the average pixel length across the center point of the wound, which was found by determining the entire length of the wound and finding the midpoint, as described previously [10]. Ten sections were obtained from the center of each wound bed, with the midline serving as an approximation for the deepest portion of the wound. The ET from five fields of unwounded, hair-bearing epidermis adjacent to the scar bilaterally were also taken as reference. The ET was expressed as a ratio relative to the unwounded epidermis.

**Collagen Deposition and Organization:** Collagen deposition and organization were visualized with the Masson’s Trichrome stain as previously described [29] and analyzed with ImageJ using digital densitometry recognition according to prior studies [10,30]. The area of interest, the neodermis, was traced manually, and collagen deposition in that region was expressed as a percentage (positive staining collagen (pixels)/total pixels ∗ 100).

### 2.9. Immunofluorescence Staining and Analysis of Tissue Sections with CD3 and K14

Tissues were fixed in 10% formalin at room temperature and then embedded in paraffin followed by sectioning into 5 mm sections. After deparaffinization and rehydration of the sections, they were immersed in heat-mediated antigen retrieval solution (10 mM sodium citrate buffer, pH 6.0) at 100 °C for 20 min. The sections were first incubated with blocking buffer (5% Goat Serum/5% Albumin in 1× PBS, pH 7.0) for one hour before incubation with anti-CD3 or anti-K14 for two hours using 5% horse serum at room temperature as previously described [10,12]. The tissue sections were then incubated with primary antibodies CD3 (1:500; Santa Cruz) or K14 (1:500; Santa Cruz) overnight at 4 °C. The sections were then washed with PBS and incubated with secondary antibodies (Alexa-fluor 488 anti-rabbit 1:1000, Alexa-fluor 568 anti-goat 1:1000; Invitrogen, Waltham, MA, USA) for one hour at room temperature. 4′-6-diamindino-2-phenyl-indole (DAPI Vectashield, Vector Laboratories, Burlingame, CA, USA) was used as a counter stain as performed in prior studies [10,12]. The sections were viewed using a Zeiss Axiovert microscope and Axiovision 4.8 software, and image analysis was conducted using ImageJ (NIH software). For each section, a randomized area (magnification 40×) was photographed and assessed for inflammatory response with CD3 and re-epithelialization with K14. All assessments were conducted blindly by two reviewers.

**Inflammatory Response:** The degree of the immune response was assessed qualitatively by two blinded reviewers using randomized field views for each CD3-stained immunofluorescent image (40×). The amount of T-cells present near the wound bed served as a measure of the total immune response [30].

**Re-Epithelialization**: Re-epithelialization was assessed qualitatively by two blinded reviewers using immunofluorescence stains of keratin-14 (K14) with randomized field views of each stained image (40×) [31,32].

### 2.10. Statistical Analysis

A graphical abstract was created using BioRender (Toronto, ON, Canada). Statistical analyses were done using the Sigma Plot software (version 11.0; Systat Software, Inc., Chicago, IL, USA). All data were measured in pixels and presented as means ± standard deviation unless otherwise stated. To determine statistical significance between treatments, a one-way analysis of variance (ANOVA) Tukey–Kramer post hoc analysis was employed. The Student’s *t*-test and Mann–Whitney tests were used for comparisons involving two experimental groups. A *p*-value of <0.05 was considered statistically significant and indicated with an asterisk in graphs and tables.

## 3. Results

### 3.1. Adipose-Derived Cells Are Viable In Vitro and Migrate within the Liquid Dermal Scaffold

**Figure 2:**
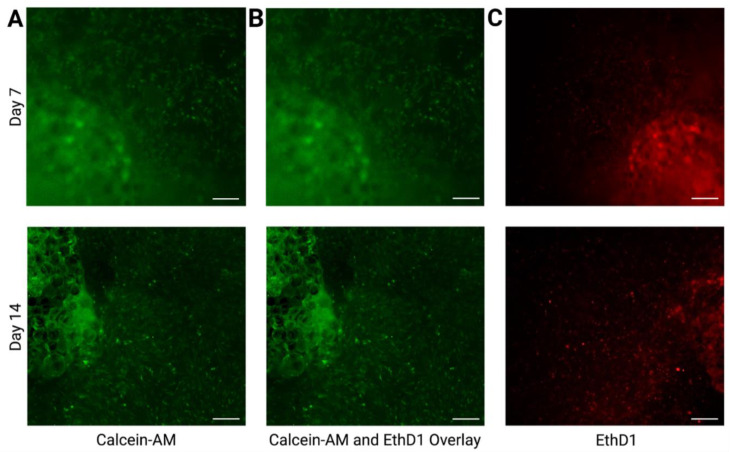
Live/Dead assay at days 7 and 14 with Calcein-AM overlay (**A**) indicating the presence of live cells in the gel, Calcein-AM, and EthD1 overlay (**B**) demonstrating negligible dead cells in the system, as well as the negative control (**C**) with EthD1 overlay demonstrating the presence of dead cells after treatment with 70% ethanol. Scale bars for (**A**–**C**) represent 150 µm.

To ensure the presence, viability, and migration of adipose-derived stem cells from adipose micro-fragments within the liquid dermal scaffold, a Live/Dead assay was preformed, demonstrating cell migration both at day 7 and day 14 within the scaffold (Figure 2A), with Calcein-AM showing live cells and EthD1 indicating dead cells in the system. There were negligent dead cells in the Calcein-AM-EthD1 overlay, demonstrating that the liquid dermal scaffold is non-toxic to cells (Figure 2B). In Figure 2C, the negative control demonstrates the presence of dead cells after the samples were bathed in 70% ethanol.

### 3.2. Adipose Micro-Fragments May Accelerate Wound Closure

**Figure 3:**
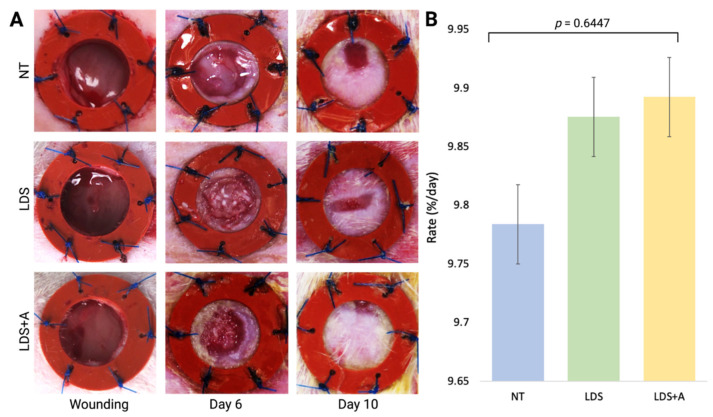
Wound planimetry assessment of full-thickness splinted wounds treated with either no treatment control (NT), a liquid dermal scaffold alone (LDS), or liquid dermal scaffold with adipose micro-fragments (LDS+A). (**A**) Wound healing across treatment conditions on the day of wounding, day 6, and day 10 is shown. (**B**) Differences in the rate of wound closure (%/day) is depicted with results expressed as mean ± standard error, with *n =* 4 across treatments.

Wound closure progression was observed throughout the experiment with photographs acquired at two different timepoints, day 6 and day 10 (Figure 3A), with the rate of wound closure (H(3) = 0.8781, *p* = 0.6447) demonstrated in Figure 3B. While not statistically significant, both the LDS and LDS+A treatments had similar closure rates with 2D analysis which were marginally increased compared to the NT control ($\bar{𝑥}$ = 9.88 ± 0.11, $\bar{𝑥}$ = 9.89 ± 0.16, $\bar{𝑥}$ = 9.78 ± 0.21 for LDS, LDS+A, and NT, respectively).

### 3.3. Treated Wounds Have Thicker Epidermis and Enhanced Re-Epithelialization

**Figure 4:**
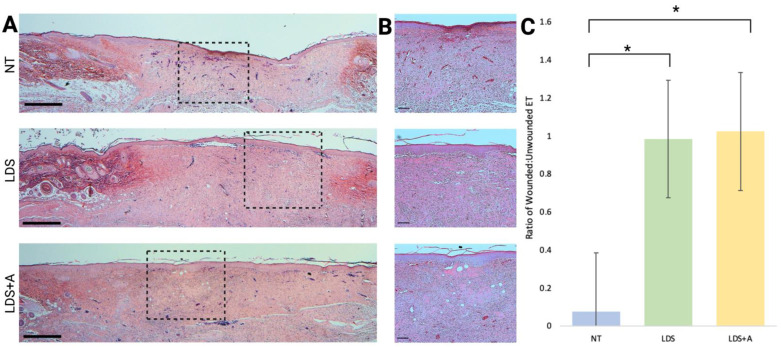
A histological evaluation of wound tissue using H&E staining. (**A**,**B**) Representative histological analysis used to quantify epidermal thickness is shown with the wound bed at 2× magnification (**A**) and 10× magnification (**B**) for the NT, LDS, and LDS+A treatment conditions. Black dashed rectangles show the wound areas demonstrated at higher magnification on the right. Scale bars for (**A,B**) represent 100 µm. (**C**) The mean ratio of wounded-to-unwounded epidermis is displayed. Using H&E-stained slides, the ET of each wound was measured from the center of the wound area and then compared to the mean of 5 measurements of adjacent, unwounded tissue from the same sample. The results are expressed as mean ± standard error, *n* = 4 for all treatments, * *p* < 0.001.

Five measurements of epidermal thickness (ET) from unwounded skin at the periphery of each sample were averaged and compared to the ET at the center of the wound, and a ratio of the wounded:unwounded epidermis was obtained. It was found that the treatment condition had a significant effect on the rate of epithelialization (*p* < 0.001, Figure 4C). The wounds with LDS alone and LDS+A had statistically significant larger epidermal thickness ratios compared to the control ($\bar{𝑥}$ = 0.984 ± 0.022, $\bar{𝑥}$ = 1.024 ± 0.063, $\bar{𝑥}$ = 0.076 ± 0.154 for LDS, LDS+A, and NT, respectively, *p* < 0.001). The two experimental treatments were comparable (Figure 4C). No epidermis was observed at the center of the no treatment wounds. Representative samples demonstrating ET with H&E staining are provided at 2× and 10× magnification (Figure 4A,B).

**Figure 5:**
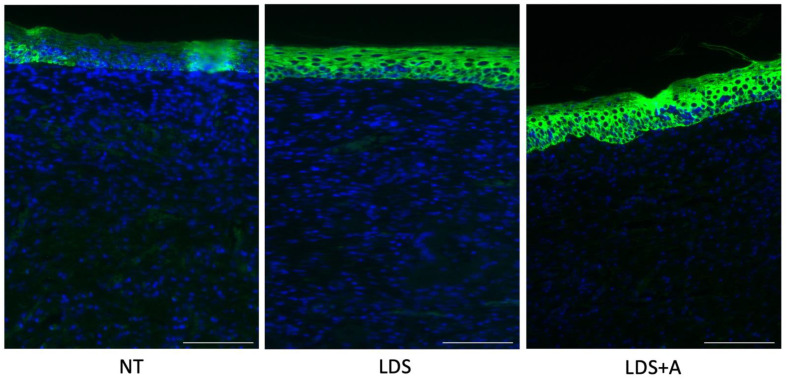
Re-epithelialization as measured by keratin-14. Immunofluorescence staining of K14 (green) with nuclear staining using DAPI (blue) for the NT, LDS, and LDS+A treatment conditions. Scale bars represent 50 µm.

Immunofluorescence staining of keratin-14, a marker of re-epithelialization [31,32], revealed that the LDS and LDS+A treatment groups demonstrated increased keratinization relative to the NT control which did not have keratinization over the wound bed, thus indicating improved re-epithelialization in the treatment conditions (Figure 5). The difference between the LDS and LDS+A groups was negligible.

### 3.4. Wounds Treated with LDS+A Had the Highest Collagen Content

**Figure 6:**
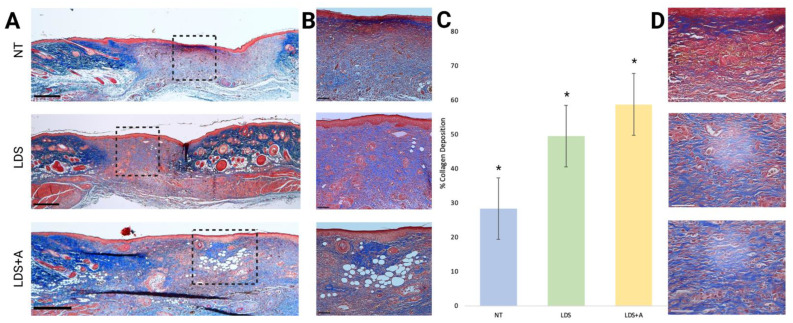
Analysis of collagen deposition in wound tissue. Histological analysis with Masson’s Trichrome staining is shown for the whole wound at 2× magnification (**A**) and at 10× magnification (**B**) for NT, LDS, and LDS+A treatments. Black dashed rectangles show the wound areas demonstrated at higher magnification on the right. Scale bars for (**A,B**) represent 100 µm. (**C**) The percentage of collagen deposition across the wound bed for all treatment groups is displayed. The results are expressed as mean ± standard error, *n* = 4 for all treatments, * *p* < 0.001. (**D**) The wounds at 40× magnification are shown for all treatments with scale bars representing 50 µm.

The total percentage of collagen deposition was used as a measure of granulation tissue and matrix formation. This was done by analyzing Masson’s Trichrome-stained sections of wound tissue with Image J. Representative samples are provided at 2× and 10× magnification, respectively (Figure 6A,B). A significantly larger area was occupied by positive collagen staining in the LDS+A and LDS only treatments compared to the NT control ($\bar{𝑥}$ = 761,935.8 ± 23,156.9, $\bar{𝑥}$ = 626,181.3 ± 27,095.9, and $\bar{𝑥}$ = 382,071.8 ± 26,574.0 respectively, *p* < 0.001) (Figure 6C). However, a significant difference was also observed between the LDS+A and LDS-only treatments, with a larger area being occupied by the LDS+A wounds (*p* = 0.0003). The wounds were further examined at 40× magnification (Figure 6D) for patterns of collagen deposition. The majority of LDS and LDS+A-treated wounds demonstrated mature collagen deposition with an organized and reticular collagen pattern compared to that of the NT-treated wounds, which demonstrated immature collagen deposition with disorganized, wavy collagen bundles and large parallel bundles formed perpendicular to the basement membrane (Figure 6D). However, no clear differences could be established in the collagen pattern deposition between the LDS and LDS+A groups.

Further, clear bubbles are present within the LDS+A images, which are most likely remnants of adipose micro-fragments which were washed out during the staining processes.

### 3.5. Control Wounds Had a Greater Immune Response

**Figure 7:**
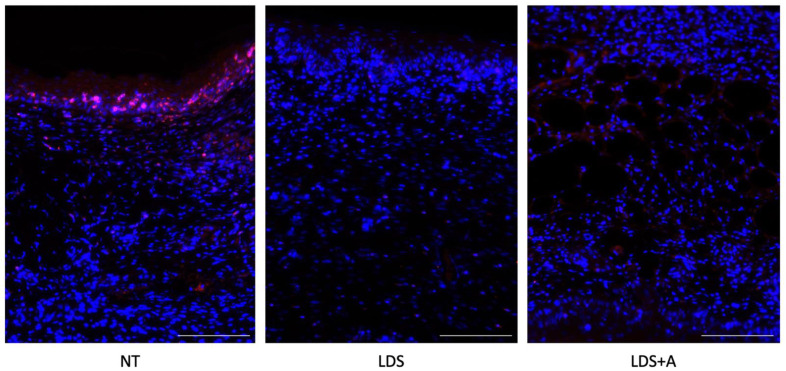
Immunofluorescence staining for CD3. CD3+ cells in red, cell nuclei stained with DAPI in blue for the NT, LDS, and LDS+A treatment conditions. Scale bars represent 50 µm.

To assess the immune response as well as T-lymphocyte infiltration and persistence in healed wounds, immunofluorescence (IF) staining with an anti-CD3+ pan T-cell marker was used. LDS and LDS+A groups demonstrated significantly reduced, if not entirely absent, infiltrated immune cells compared to the NT control (Figure 7). A qualitative assessment of the stains showed that the wounds in the NT control had more T-cells present near the wound bed and forming epidermis, whereas in the LDS+A treatment, scarce T-cells were present in the dermis surrounding adipose fragments (Figure 7).

## 4. Discussion

There are vast benefits to be ascertained from the technological advancements in wound healing at small and large scales, both promoting individualized care and a better quality of life for patients, as well as relieving economic burden institutionally [7,8,9]. As wound healing is an intricate and arduous process that can be easily disrupted with any deviation within the healing progression, our group previously developed a collagen-GAG-based LDS capable of phase change in vivo from the powder to gel form, which we previously demonstrated is non-toxic to human fibroblasts [12]. Additionally, we showed that this scaffold accelerates and ameliorates wound healing in vivo, improving outcomes in both a hypertrophic scar animal model [12] as well as a diabetic murine model [10], mimicking delayed healing. Further studies established that this liquid scaffold is compatible with adipose-derived stem cells and accelerates wound closure with better outcomes in a delayed wound healing model [10].

The in situ-forming nature of this scaffold is an essential feature of chronic and burn wound treatment. It circumvents many of the shortcomings of the commercially available products, including solid scaffolds and engineered skin substitutes [12,33]. Notably, in contrast to these other products, our scaffold is liquid and flowable, and thus able to conform to wounds of any shape and depth from the bottom-up, thereby eliminating regions where cells must migrate during the wound repair process [10,11,34]. Further, the longer a biological wound remains open, the probability of hypertrophic scarring and susceptibility to infection increases [12,33,35]. Thus, our scaffold’s ability to accelerate wound healing compared to standard treatment protocols further circumvents these issues [10,12,36].

Another issue of hard-to-treat wounds is the paucity of progenitor cells [4]. The use of mesenchymal stem cells has demonstrated promising findings as these multipotent cells can differentiate into numerous cell types such as fibroblasts, keratinocytes, epithelial cells, and endothelial cells [37] and secrete essential growth factors which promote wound healing, namely increasing angiogenesis and cell proliferation and decreasing the inflammatory and immune responses of the wound [10,12,21]. Adipose tissue is a rich source of mesenchymal stem cells with its use growing in popularity for both regenerative and cosmetic purposes [21,22,23]. Another advantage of adipose tissue is that it can be harvested repeatedly in a minimally invasive manner using procedures such as liposuction [38]. A previous study using adipose-derived stem cells (ASC) in combination with our LDS demonstrated that the ASCs could maintain their viability within the scaffold and were found within the wounded area after re-epithelialization [10]. Additionally, using ASC demonstrated improved tissue quality [10]. However, studies have shown that the use of ASC has limitations, particularly regarding their engraftment and long-term survivability [39]. Moreover, there are many practical limitations in using transplanted ASCs, including determining the optimal source of adipose, as well as processing and administering the stem cells clinically [39]. In this study we sought to investigate this further, instead using adipose micro-fragments as a viable source of progenitor cells. Our results suggested that the application of adipose micro-fragments in combination with our LDS is a feasible approach for ameliorating healing progression and maturation in a murine model.

In vitro investigations of the LDS+A combination demonstrated that cells do migrate out of the adipose micro-fragments and are viable within the system on both days 7 and 14 of the experiment, thus demonstrating that the use of micro-fragments is a feasible source of progenitor cells to be used in conjunction with the LDS. Future studies should incorporate scratch assays to quantify cell migration; moreover, further experiments are required to elucidate the cell types migrating out of the micro-fragments, in order to better understand how the LDS+A combination is involved in wound healing physiology.

In vivo, it was found that the addition of adipose micro-fragments did not alter wound closure rates in our study compared to the LDS alone or NT groups. However, no significant differences were found between any treatment group. This contradicts previous findings which have demonstrated accelerated wound healing with LDS alone in murine models [10,12,36]. One potential confounding factor could be the varying size of adipose micro-fragments within the treatment, as a standardization of size was not possible with the preparation technique used. However, this discrepancy with past studies is mainly thought to be a consequence of a small sample size, as it was observed that the LDS+A and LDS groups similarly required fewer days for epithelialization and had more fully closed wounds by day 10 compared to the NT group. This was further corroborated by histological findings which show more mature stages of wound healing in the LDS+A and LDS groups compared to the NT control. Additionally, wounds treated with LDS+A and LDS alone were found to have more shallow-appearing wounds; however, this was not quantified due to inaccuracies obtained with standard 2D photographs [10,40]. Conversely, this depth difference could be rendered irrelevant given that wound contraction is the primary healing mechanism in murine models [41,42], rather than epithelialization and granulation tissue formation observed in human wound healing [25]. Nonetheless, this was controlled for in our study using splints to prevent wound contracture. Future studies should take these into consideration using larger sample sizes and 3D modeling of wounds for depth and volume analysis [40] to elucidate the importance of the results that were not found to be of significance in this study and to further evaluate wound closure rates which, as previously mentioned above, can be quite advantageous in limiting complications in the wound repair process if accelerated [4].

Despite the lack of an observed increase in the wound healing rate, we were able to detect changes which illustrate a superior wound healing progression and maturation in the LDS+A and LDS groups compared to the NT control. The healed wounds with LDS+A and LDS had a greater epidermal thickness and demonstrated enhanced re-epithelialization, as well as more mature and organized patterns of collagen deposition; however, future studies should incorporate methods to quantify these collagen patterns, such as using birefringence.

Moreover, it was found that the LDS+A group had the highest collagen content in its neodermis. Other studies have found similar increases in collagen content using different animal wound models; however, these have used direct ASC injection rather than adipose micro-fragments, such as in delayed-healing murine models [10,42]. This has been theorized to be due to either direct fibroblast stimulation by adipose-derived stem cells to increase procollagen gene expression [43] or indirectly via differentiation of ASCs into fibroblasts [44]. This increase in collagen suggests that granulation tissue is forming rather than scar tissue [42]; however, future studies should examine specific collagen types and protein expression to elucidate this further.

Additionally, reduced immune cell infiltration, specifically of T-lymphocytes, was noted in the LDS+A and LDS groups compared to the NT control. Previous studies demonstrated the immunosuppressive tendencies of ASC [45] which are beneficial in wound healing as immune cells release cytokines and other inflammatory factors which can lead to a dysregulation of acute wound healing, leading to the formation of chronic wounds [46]. Further, a prolonged inflammatory phase leads to delayed healing, which is often of poor quality [47]. This can be attributed to an overabundance of inflammatory mediators, an inability of macrophages to clear cellular debris, and the release of metalloproteinases by chronic wound macrophages (MMP-2 and MMP-9) which degrade ECM and prevent proliferative healing [47]. It should be noted that some T-lymphocytes were seen around the adipose micro-fragments. This can potentially be explained by the use of allogenic adipose micro-fragments. Future studies should examine this response in more detail to see if it impacts healing progression, further exploring immune response with autogenic adipose.

Apart from the aforementioned challenges and limitations, another consideration to note is that our study was conducted using a healthy murine model and was thus under the assumption that the NT wounds underwent normal wound healing physiology. Future investigations are warranted using models of aberrant wound healing to note if there are any further differences noted between treatment groups. However, as aforementioned, rodent wound healing differs from that of humans. A porcine wound model would be the best mimic of human skin given its biological similarities including thickness of the layer, blood vessel density, dermal collagen, elastin, and hair follicles [48]. Sample size is another factor that might have limited the discrete findings, so future studies using a greater study sample will increase the statistical power and improve the generalizations of our findings. These considerations will garner further evidence to propel the clinical transition of this scaffold.

## 5. Conclusions

This study demonstrates the application of adipose micro-fragments within a non-rejectable in situ liquid dermal scaffold for the amelioration of wound healing in a healthy murine model. The addition of adipose micro-fragments demonstrated improved tissue quality and may provide a promising alternative to our previously developed liquid scaffold. This combination has therapeutic potential for use in large, full-thickness wounds.

## Data Availability

The data that support the findings of this study are available from the corresponding author upon request.
